# Identification of BCL3 as a biomarker for chondrocyte programmed cell death in osteoarthritis

**DOI:** 10.1111/iep.12522

**Published:** 2024-12-16

**Authors:** Junxiao Ren, Rui Li, Chen Meng, Yongqing Xu, Chuan Li

**Affiliations:** ^1^ Yunnan University of Chinese Medicine Kunming Yunnan China; ^2^ Kunming Medical University Kunming Yunnan China; ^3^ The 920th Hospital of Joint Logistics SupportForce of PLA Kunming Yunnan China; ^4^ Engineering Laboratory of Peptides of Chinese Academy of Sciences Kunming Yunnan China

**Keywords:** BCL3, biomarker, chondrocyte, osteoarthritis, programmed cell death

## Abstract

Osteoarthritis (OA) is a condition that is widely prevalent and causes joint pain and disability, with programmed cell death (PCD) playing a role in its pathogenesis. This study aimed to identify biomarkers associated with PCD in OA and explore their potential roles. Three RNA‐sequencing datasets (GSE114007, GSE51588 and GSE220243) related to OA were analysed. Differential expression and weighted gene co‐expression network identified key differentially expressed PCD‐related genes (DE‐PRMGs). Potential biomarkers were identified and validated through receiver operating characteristic (ROC) curves, correlation analyses, gene set enrichment analysis, single‐cell expression and RT‐qPCR. A total of 45 DE‐PRMGs were identified, affecting pathways like TNF signalling and RNA degradation. BCL3, TREM2 and NRP2 were prioritized as potential OA biomarkers, which are associated with ribosome function and immune cell infiltration and potentially linked to PCD. The functional role of one of the molecules identified, BCL3, was explored further using a cell model of inflammation induced chondrocytes. BCL3 was significantly down regulated in OA samples from the public dataset and clinical samples analysed by RT‐qPCR. BCL3 overexpression reduced apoptosis in chondrocytes stimulated with inflammatory cytokines. Thus the functional studies highlighted the anti‐apoptotic role of BCL3 in chondrocytes and provide new insights into OA pathogenesis with potential for future therapeutic development.

## 1 INTRODUCTION

Osteoarthritis (OA) is a common condition that predominantly affects individuals in the middle‐aged and older population. Symptoms in the initial stages of the disease include pain and stiffness in the knee, hip, hand and other joints.^1^, ^2^ As the condition progresses, in severe instances joint movement may become restricted or deformed over time. A comprehensive study conducted by the Harvard School of Public Health and the World Health Organization, spanning 30 years and involving 195 countries and regions, has revealed concerning statistics regarding knee and hip arthritis.^3^ Currently, an estimated 303 million individuals worldwide are struggling with this incapacitating condition. Even more troubling, approximately 15 million new cases of OA are diagnosed each year, imposing a substantial burden on global healthcare systems.^4^, ^5^ Comparing to the early 1990s, there has been a 9.3% increase in the number of patients with OA. With the ageing population and other societal shifts, the prevalence of OA in China has surged from 26.1 million three decades ago to 61.2 million today, equating to a rate of 3–4 individuals per 100 Chinese citizens.^6^ This has a profound social and economic impact on the physical and mental well‐being of patients, as well as their overall quality of life.^7^, ^8^ It is critical to investigate and elucidate the molecular basis of OA progression, with the goal of identifying potential predictive markers and therapeutic targets.

Programmed cell death (PCD) is a fundamental biological process present across various organisms, playing crucial roles in growth, development, tissue repair and disease pathogenesis.^9^ The term PCD encompasses several different processes including apoptosis, necroptosis, programmed necrosis and ferroptosis.^10^ The occurrence of PCD involves intricate cascades of signalling reactions mediated by multiple effector molecules, exhibiting unique biochemical characteristics, morphological features and immunological consequences.^11^ It has been well‐documented that inflammatory factors constitute a fundamental basis for the initiation and progression of PCD, acting as a major inducer of OA development.^12^, ^13^ The apoptosis of chondroytes has been recognized as a major event which leads to cartilage degeneration in OA.^14^, ^15^ However, there is a lack of systematic investigations elucidating the biomarkers of PCD related to the pathogenesis of OA.

This study aims to systematically identify and characterize potential biomarkers associated with PCD in OA pathogenesis. Through the analyses of multiple datasets comprising osteoarthritic and normal cartilage samples, key PCD‐related genes were prioritized as candidate biomarkers through differential expression, co‐expression network analysis and machine learning algorithms. The diagnostic value of these biomarkers was evaluated, together with their associations with immune infiltration and expression patterns across single‐cell population. We also explored the functional role of BCL3 in inflammation‐induced chondrocyte cell model. Our integrative approach coupled with experimental validation uncovered BCL3 as a biomarker indicative of the role of a PCD mechanism underlying osteoarthritis. These observations suggest its potential clinical applications, and provide insights into disease biology for therapeutic development.

## 2 MATERIALS AND METHODS

### 2.1 Public data retrieval

RNA‐sequencing data (GSE114007 and GSE51588) and single‐cell RNA‐seq data (GSE220243) related to OA were obtained from the Gene Expression Omnibus (GEO) database, accessible at http://www.ncbi.nlm.nih.gov/geo/. GSE114007 consisted of 18 OA cartilage tissue and 20 normal cartilage tissue samples, serving as the training set. GSE51588 (OA:normal = 40:10) was used as the testing set, while GSE220243 included six OA cartilage tissue and six normal cartilage tissue samples. Additionally, 3659 PCD‐related genes (PCD‐RGs) were retrieved from a previously published literature.^16^

### 2.2 Differential expression analysis

Differential expression analysis of OA and normal samples was conducted using the ‘limma’ package (v 3.54.2).^17^ The thresholds of adj.P1 were applied to identify differentially expressed genes (DEGs). Volcano plots and heatmaps were generated with the ‘ggVolcano’ (v 0.0.2) and ‘ComplexHeatmap’ (v 2.14.0) packages to visually represent the results of the analysis.^18^

### 2.3 Weighted gene co‐expression network analysis (WGCNA)

WGCNA method was employed to determine the essential modular genes associated with both OA and normal phenotypic traits. Initially, sample clustering was conducted to eliminate any potential outliers within the data set. Subsequently, a soft threshold, referred to as power, was established. Following this, gene dissimilarity coefficients were computed using the neighbour‐joining approach to develop a systematic clustering tree. To ensure the robustness of the gene modules, a minimum gene count per module (minModuleSize) of 50 was set based on the requirements of the hybrid dynamic shear tree. Moreover, a mergeCutHeight of 0.3 (equivalent to 70% similarity) was implemented to merge modules that exhibited similar characteristics as identified by the dynamic shear tree algorithm. Lastly, the associations between the phenotypic traits and gene modules were examined to ascertain the pivotal modules and their corresponding essential genes within these modules.

### 2.4 Identification and functional annotation of differentially expressed‐PCD‐related module genes (DE‐PRMGs)

The DE‐PRMGs were identified by overlapping DEGs, PCD‐RGs and key module genes through the ‘ggVennDiagram’ package (v 1.2.2). Subsequently, GO and KEGG enrichment analyses were conducted on the DE‐PRMGs using the ‘clusterProfiler’ package with a significance threshold of *p* < .05 (v 4.6.2).^19^ The results of the enrichment analyses were then visualized using the ‘treemap’ package (v 2.4–4).^20^ Additionally, PPI networks for the DE‐PRMGs were constructed using the STRING website, with an interaction score cutoff of 0.4, and visualized using Cytoscape software (v 3.8.2).^21^ Finally, the DE‐PRMGs with the top 20 scores from the MCC algorithm in the Cytoscape plugin cytoHubba were selected as candidate genes for further analysis.

### 2.5 Signature gene identification

The least absolute shrinkage and selection operator (LASSO) regression was performed on candidate genes using the ‘glmnet’ package (v 4.1–4) to identify signature genes.^22^ Subsequently, the signature genes were further filtered using the XGBoost algorithm with the ‘xgboost’ package (v 1.7.5.1). The key signature genes were determined by identifying the intersection of signature genes obtained from both algorithms.

### 2.6 Identification and functional annotation of biomarkers

The diagnostic ability of the key signature genes in the training and testing sets was assessed by generating ROC curves with the ‘pROC’ (v 1.18.0) package. Biomarkers were chosen based on AUC values above 0.7 in both datasets.^23^ To explore the relationships between biomarkers, a thorough analysis was conducted using various statistical methods. Initially, Spearman correlation was used to identify correlations among the biomarkers in the training set. Furthermore, Pearson correlation coefficients between the biomarkers and the expression levels of all genes in the training set were calculated. These coefficients were then ranked in descending order to reveal the strongest associations.

The ‘clusterProfile’ package was employed for gene set enrichment analysis (GSEA‐KEGG) in order to better understand the functional implications of the biomarkers. To address multiple testing concerns, we utilized the false discovery rate (FDR) method for adjusted p‐values. This allowed us to explore the potential significance of the biomarkers and their associations. A p.adjust value below 0.05 was considered significant. To validate our results, we compared biomarker expression levels between OA and normal samples in the training set using the ‘ggplot2’ package (version 3.3.6).^24^ This comparison was further validated in an independent testing set to ensure result reliability. Our goal was to gain a comprehensive understanding of the biomarkers' relationships and significance in the context of OA. To investigate the impact of m6A on the translational stability of biomarkers in OA, we obtained biomarker sequence files from NCBI and predicted m6A binding sites using SRAMP (http://www.cuilab.cn/sramp/).

### 2.7 Immune infiltration analysis

To investigate the relationship between biomarkers and immune cells, we utilized the ‘GSVA’ package (version 1.42.0) to quantify the presence of 28 immune cell infiltrations in both OA and normal samples within the training set.^25^ This quantification was performed using the ssGSEA algorithm with the goal of understanding the correlation between these immune cell populations and potential biomarkers. Comparative analysis of immune cells in individuals with OA versus those without the condition was carried out. Furthermore, correlations between biomarkers and immune cells that showed significant differences were analysed using Spearman analysis.

### 2.8 Construction of regulatory network

To explore the regulatory network of biomarkers, a competing endogenous RNA (ceRNA) regulatory network was constructed. The miRnet database was employed for predicting miRNAs and lncRNAs, while the Cytoscape software (v 3.8.2) was used to establish the ceRNA regulatory network. Transcription factors (TFs) associated with biomarkers were predicted through NetworkAnalyst to build a TF‐biomarker regulatory network. Furthermore, drug‐biomarker regulatory networks were formed by predicting biomarker‐related drugs using the DrugBank database.

### 2.9 Single‐cell analysis

To investigate the potential impact of PCD on the occurrence of OA, we analysed biomarker expression in various cell types using the GSE220243 dataset. The scRNA‐seq dataset GSE220243 underwent quality control with the ‘Seurat’ package (v 4.1.0) based on specific criteria: (1) exclusion of cells with less than 100 or more than 6000 expressed genes; (2) exclusion of cells with less than 200 genes or covered by less than 3 cells; (3) inclusion of cells with mitochondrial gene proportion below 20%.^26^ The functions ‘NormalizeData’ and ‘FindVariableFeatures’ identified the top 2000 most variable genes. Subsequently, significant principal components were determined using ‘ScaleData’ and ‘JackStrawPlot’. Cell clustering analysis followed dimensionality reduction via the UMAP method (resolution = 0.6), and the cell types were annotated using the previously published references.^27^ The proportion of annotated cells in OA and normal samples was visualized using the ‘ggplot2’ package. Differential marker genes between annotated cells were identified using the Wilcoxon test. Expression levels of biomarkers in annotated cells were compared between OA and normal groups with the Wilcoxon test (*p* < .05). Cell communication analysis was conducted using ‘CellChat’ (*p* < .05).

### 2.10 Real‐time quantitative‐polymerase chain reaction (RT‐qPCR)

A total of 10 frozen tissue samples were collected from individuals at the 920th Hospital with approval from the 920th Hospital Ethics Committee of the Joint Logistic Support Force (No. 2023‐124‐01). The samples, consisting of five normal and five OA tissues, were handled following guidelines and regulations, with donor consent. Total RNA was extracted from a 50 mg tissue sample using TRIzol and converted to cDNA through a reverse transcription reaction (SureScript First‐strand cDNA synthesis‐kit, Themo Fisher Scientific, CA, USA). Target gene sequences were retrieved from the National Center for Biotechnology Information (NCBI) database, and specific primers were designed using Primer‐BLAST tool (https://www.ncbi.nlm.nih.gov/tools/primer‐blast/) for RT‐qPCR amplification.^28^ RT‐qPCR analysis was conducted using SYBR Green qPCR Master Mix (Takara, Dalian, China), and the amplification was carried out on a CFX Connect real‐time quantitative fluorescent PCR instrument (Bio‐Rad Laboratories, Inc. CA, USA). The 2^−△△CT^ approach was employed to determine the relative expression levels of targets using GAPDH as the reference gene. The primer sequences are listed in Table 1.

**TABLE 1:** The forward and reverse primers.

Primer	Sequences
NRP2 F	GTTACCCTCAACAGGGAGGC
NRP2 R	CAGGAGTTGGATGACGACTGT
BCL3 F	TGAGGAGAGACTGGCACCAA
BCL3 R	ACAGTCCTCTCTCCCATCCC
TREM2 F	CCCATGATGCGGGTCTCTAC
TREM2 R	CTCAGCCCTGGAGATGCTGT
GAPDH F	CGAAGGTGGAGTCAACGGATTT
GAPDH R	ATGGGTGGAATCATATTGGAAC

### 2.11 Cell culture and treatment

The CHON‐001 human chondrocyte cell line (Cell Bank of Chinese Academy of Sciences Typical Culture Collection Center, Shanghai, China) was cultivated in Dulbecco's Modified Eagle's Medium (DMEM)/F‐12 (Gibco, Waltham, MA, USA) containing 10% fetal bovine serum (FBS; Gibco) and 1% penicillin/streptomycin (Gibco) at 37°C with 5% CO_2_. For inflammatory stimulation, CHON‐001 cells were treated with 10 ng/mL recombinant human IL‐1β and 10 ng/mL recombinant human TNF‐α (Peprotech, NJ, USA) for 48 h. To overexpress BCL3, BCL3 cDNA was cloned into pcDNA.3.1 vector (pcDNA3.1‐BCL3), with the empty plasmid as the control. Cells were transfected with pcDNA3.1‐BCL3 or pcDNA.3.1 control vector using Lipofectamine 3000 (ThemoFisher Scientific, CA, USA) for 48 hours before further experiment.

### 2.12 CCK‐8 cell viability assay

Cell viability was assessed using the Cell Counting Kit‐8 (CCK‐8, Dojindo, Cat#CK04, Kumamoto, Japan). CHON‐001 cells were seeded at a density of 6 × 10^3^ cells/well in 96‐well plates and allowed to attach overnight. Following treatment, each well containing 200 μL of culture medium received 10 μL of CCK‐8 solution, and the plates were then incubated at 37°C for 2 h. Absorbance at 450 nm was measured using a microplate reader (BioTek Instruments, Winooski, VT, USA).

### 2.13 Cleaved caspase‐3 activity assay

The activity of cleaved caspase‐3 was assayed on the CHON‐001 cell line using the Caspase‐3 Colorimetric Assay Kit (Genostaff Co., Ltd., Tokyo, Japan). CHON‐001 cells were seeded at a density of 1 × 10^6^ cells per well in a 6‐well plate. After treatment with the experimental conditions, cells were harvested, and lysed with the provided lysis buffer and the protein concentration was determined. Two hundred micrograms of protein from each sample was then incubated with the caspase‐3 specific colorimetric substrate for 2 h at 37°C. The chromogenic p‐nitroaniline product formed was detected by measuring absorbance at 405 nm using a microplate reader. The change in absorbance was directly proportional to the caspase‐3 enzymatic activity present in the cell lysates.

### 2.14 Terminal deoxynucleotidyl transferase‐mediated dUTP‐biotin nick end labelling assay (TUNEL)

The TUNEL assay kit (Beyotime Biotechnology, Beijing, China) was utilized for detecting cell apoptosis. Cells were fixed using 4% formaldehyde and were exposed to a 3% hydrogen peroxide solution for 10 min at room temperature. This was followed by labelling with a biotin labelling solution consisting of TdT enzyme (2 μL), biotin‐dUTP (48 μL) and biotin labelling solution (50 μL) at 37°C for 60 min. Post‐washing, cells were further incubated with Streptavidin HRP working solution at room temperature for 30 min. The signal was developed using 0.2 mL of DAB colour developing solution at room temperature for 5 min.

### 2.15 Statistical analysis

Statistical analysis was conducted with GraphPad Prism Software (GraphPad Software, San Diego, CA, USA). The experiments were carried out in triplicates, and findings are displayed as mean ± SD. An unpaired Student's *t*‐test was used to compare two groups, and one‐way or two‐way ANOVA followed by Bonferroni's post hoc test was employed for multiple group comparisons. Statistical significance was defined as *p* < .05.

## 3 RESULTS

### 3.1 Identification of differentially expressed PCD‐related module genes (DE‐PRMGs) in OA

**FIGURE 1:**
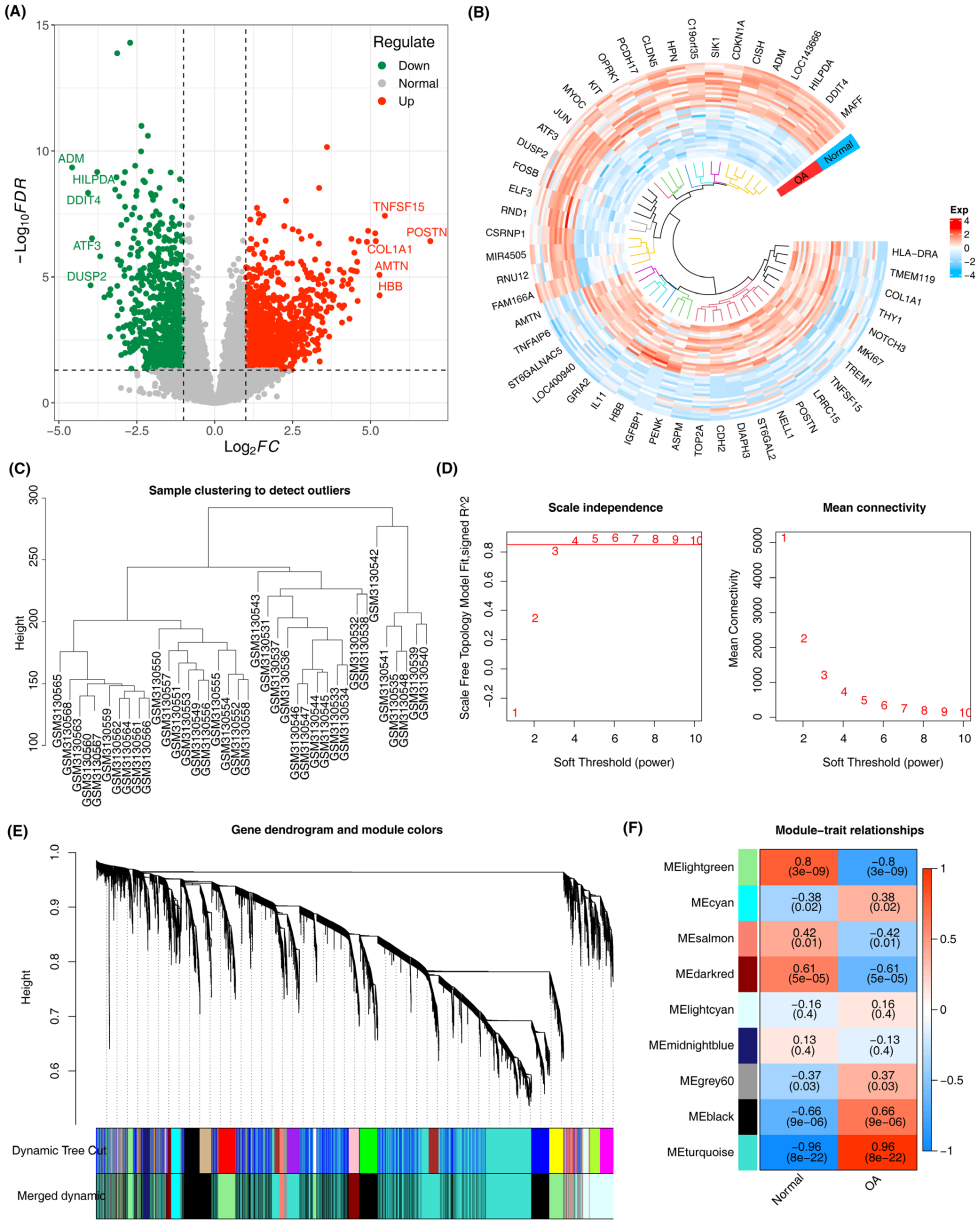
Identification of differentially expressed‐PCD‐related module genes (DE‐PRMGs) in OA. (A) Volcano plot of DEGs between OA and normal samples in GSE114007 dataset. (B) Ring heatmap of expression of top 50 DEGs in OA and normal samples. (C) Clustering of individual samples GSE114007 dataset. (D) Soft threshold filtering analysis to determine the threshold of WGCNA. (E) Module clustering tree from WGCNA analysis. Each coloured block represents a distinct module of co‐expressed genes. Red modules indicate a strong positive correlation with OA, while blue modules denote a strong negative correlation. White or lighter‐coloured modules suggest weaker or no significant correlation with OA. The numbers display the correlation coefficients, with corresponding *p*‐values in parentheses. (F) Heatmap showing the correlations of gene modules and OA traits.

Analysis of the GSE114007 dataset revealed a total of 1987 Differentially Expressed Genes (DEGs) between OA and normal samples. Of these, 1175 were upregulated and 812 were downregulated (Figure 1A,B). Within the 38 samples in GSE114007, GSM3130542 was identified as an outlier in the normal group by hierarchical clustering, and subsequently excluded from further analysis (Figure 1C). WGCNA analysis was then conducted on the remaining samples to identify gene modules associated with the OA trait. The power threshold was determined to be 4 (*R* ^2^ = 0.85) based on the position of the red line (Figure 1D). Nine modules were identified through the co‐expression network, with the turquoise module identified as the key module (|*r*| = 0.96, *p* < .001), containing 7071 key module genes (Figure 1E,F). Subsequently, 45 differentially expressed PCD‐related module genes (DE‐PRMGs) were identified by overlapping DEGs, program cell death‐related genes (PCD‐RGs) and key module genes (Figure S1A). The biological functions of DE‐PRMGs were further explored through Gene Ontology (GO) and Kyoto Encyclopedia of Genes and Genomes (KEGG) analysis. The GO analysis revealed enrichment in functions such as ‘isoprenoid biosynthetic process’ and ‘filopodium’, while the KEGG analysis showed enrichment in pathways such as the ‘Rap 1 signalling pathway’, ‘RNA degradation’ and ‘TNF signalling pathway’ (Figure S1B). These findings suggest the significance of these pathways in OA development.

### 3.2 BCL3, TREM2 and NRP2 are identified as candidate biomarkers for OA

**FIGURE 2:**
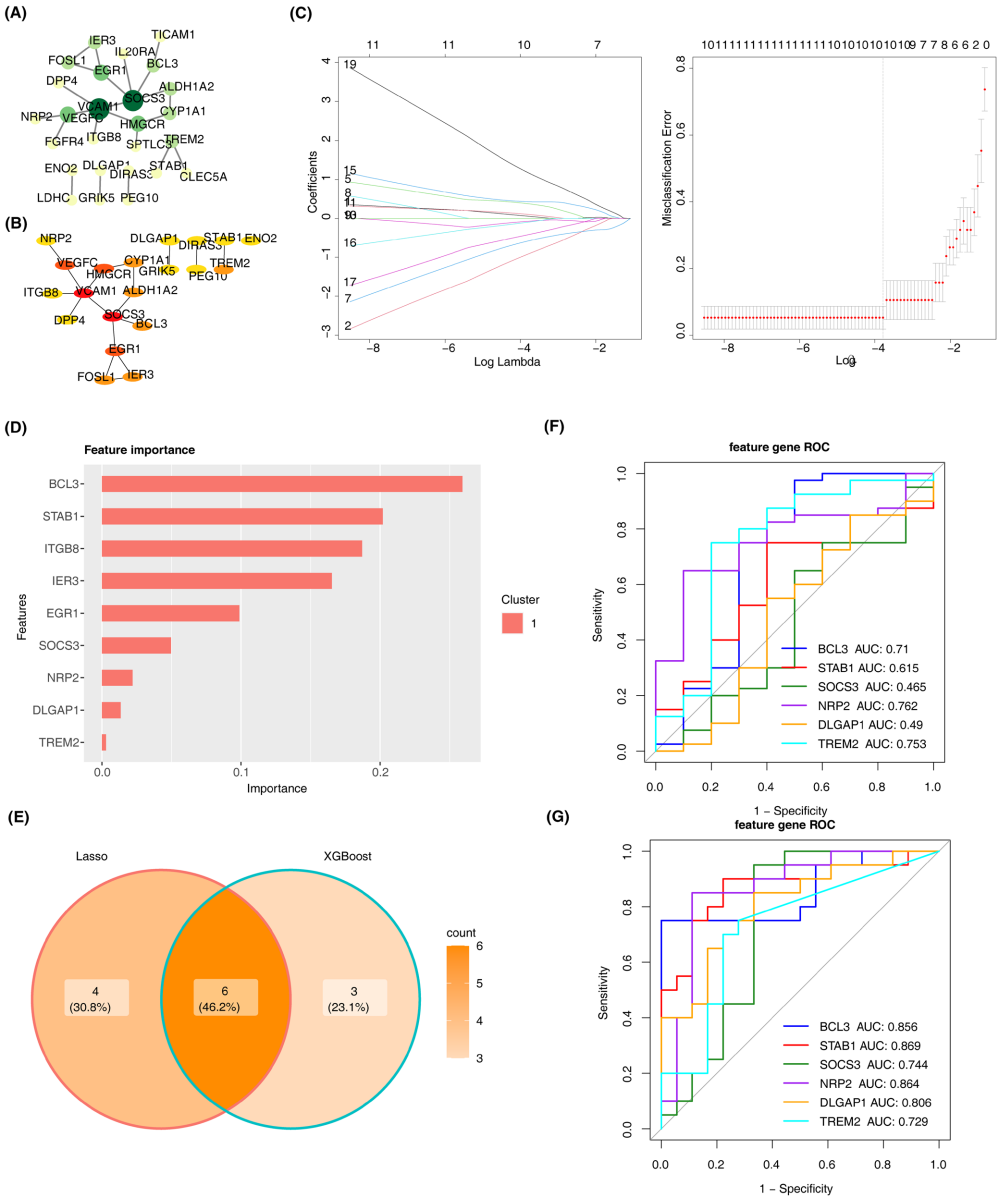
BCL3, TREM2 and NRP2 are identified as candidate biomarkers for OA. (A) PPI network of the 45 DE‐PRMGs. (B) PPI network for top 20 candidate genes. (C) LASSO regression analysis with *λ*‐selection map and logistic coefficient penalty map. (D) Important features (gene) bar chart of Xgboost analysis. (E) Venn plot of signature genes identified by LASSO regression and Xgboost. (F, G) ROC curves of signature genes in the training data GSE114007 and validation dataset GSE51588.

A protein–protein interaction (PPI) network was constructed using 45 DE‐PRMGs (Figure 2A). The top 20 DE‐PRMGs were identified as key candidate genes through the MCC scoring algorithm in Cytoscape (Figure 2B). Subsequently, LASSO regression analysis revealed ten predictor genes of high relevance to OA (VCAM1, SOCS3, HMGCR, etc.), and nine signature genes (BCL3, STAB1, ITGB8, etc.) were identified by XGBoost algorithm (Figure 2C,D). The key signature genes were obtained by the overlap of genes identified by two algorithms, which include SOCS3, BCL3, TREM2, NRP2, DLGAP1 and STAB1 (Figure 2E). Finally, ROC curves were plotted for the key signature genes in the training dataset (GSE114007) and the validation set (GSE51588). Notably, BCL3, TREM2 and NRP2 exhibited area under curve (AUC) values greater than 0.7, suggesting their potential utility as biomarkers (Figure 2F,G).

### 3.3 BCL3, TREM2 and NRP2 expression levels were associated with changes in ribosome gene programs

**FIGURE 3:**
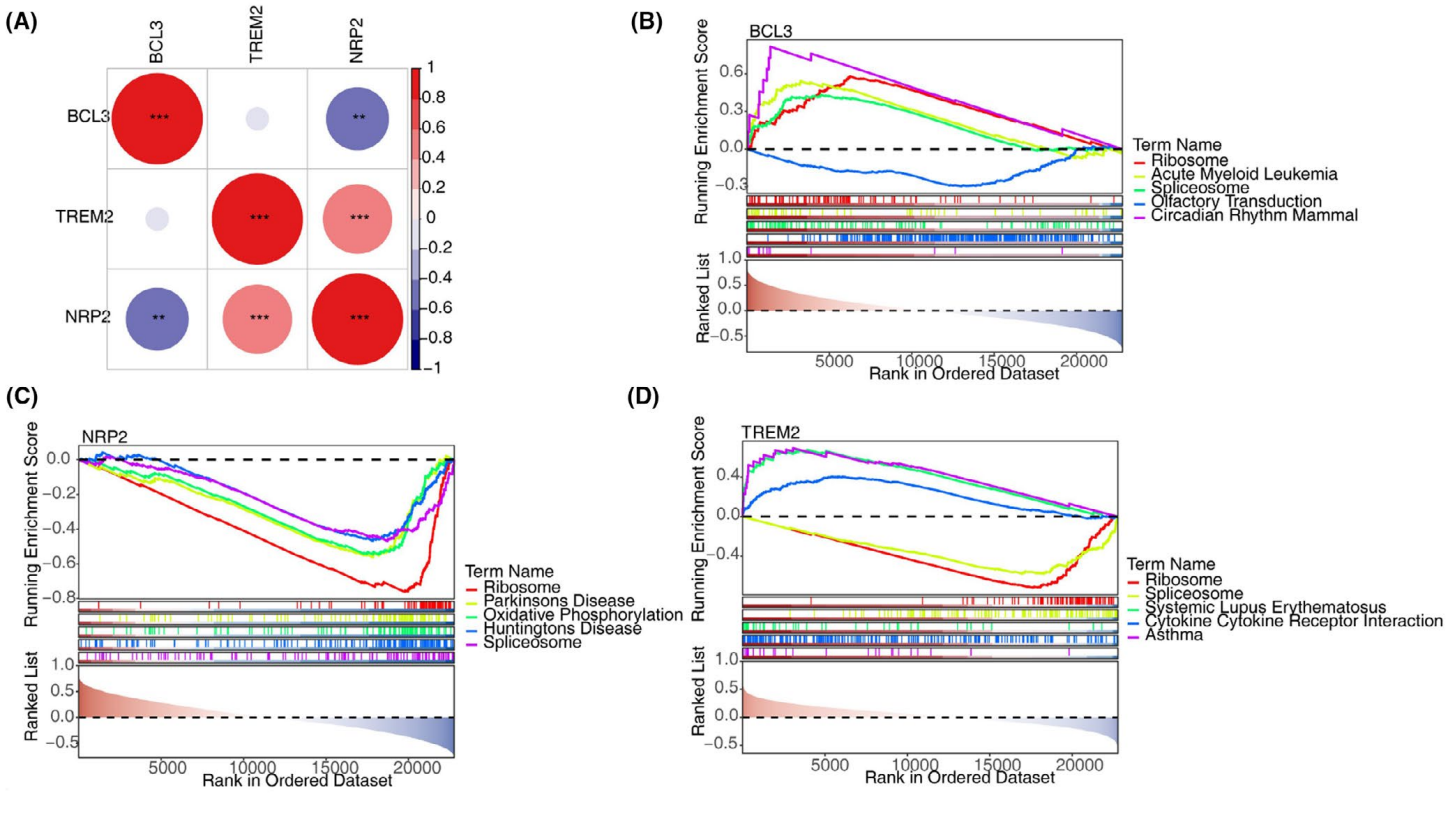
BCL3, TREM2 and NRP2 expression levels were associated with ribosome gene programs. (A) Heatmap showing the correlations between BCL3, TREM2 and NRP2 expression levels. (B–D) GSEA enrichment results for BCL3, TREM2 and NRP2, based on the expression levels of each gene (low‐expression and high‐expression samples in GSE114007). Top 5 enriched gene sets were shown.

Next, we analysed the correlation between BCL3, TREM2 and NRP2 expression levels. There was a significant negative correlation between BCL3 and NRP2 (*r* = −0.474, *p* < .05) and a positive link between TREM2 and NRP2 (*r* = 0.576, *p* < .05) (Figure 3A). To delve into the biological insights of these genes, we performed GSEA based on the expression levels of each gene (low‐expression and high‐expression samples in GSE114007). GSEA results showed that high BCL3 expression level was associated with upregulation of ribosome genes, while low expression of TREM2 and NRP2 was linked with upregulation of ribosome genes (Figure 3B–D). Our analysis revealed an association between BCL3, TREM2 and NRP2 expression and changes in ribosome gene expression. However, this relationship may be indirect, possibly stemming from broader cellular stress responses. Further research is needed to elucidate the precise mechanisms underlying these observations.

### 3.4 Immune cell infiltration correlation with BCL3, TREM2 and NRP2 expression, and the potential regulatory mechanisms of these biomarkers

**FIGURE 4:**
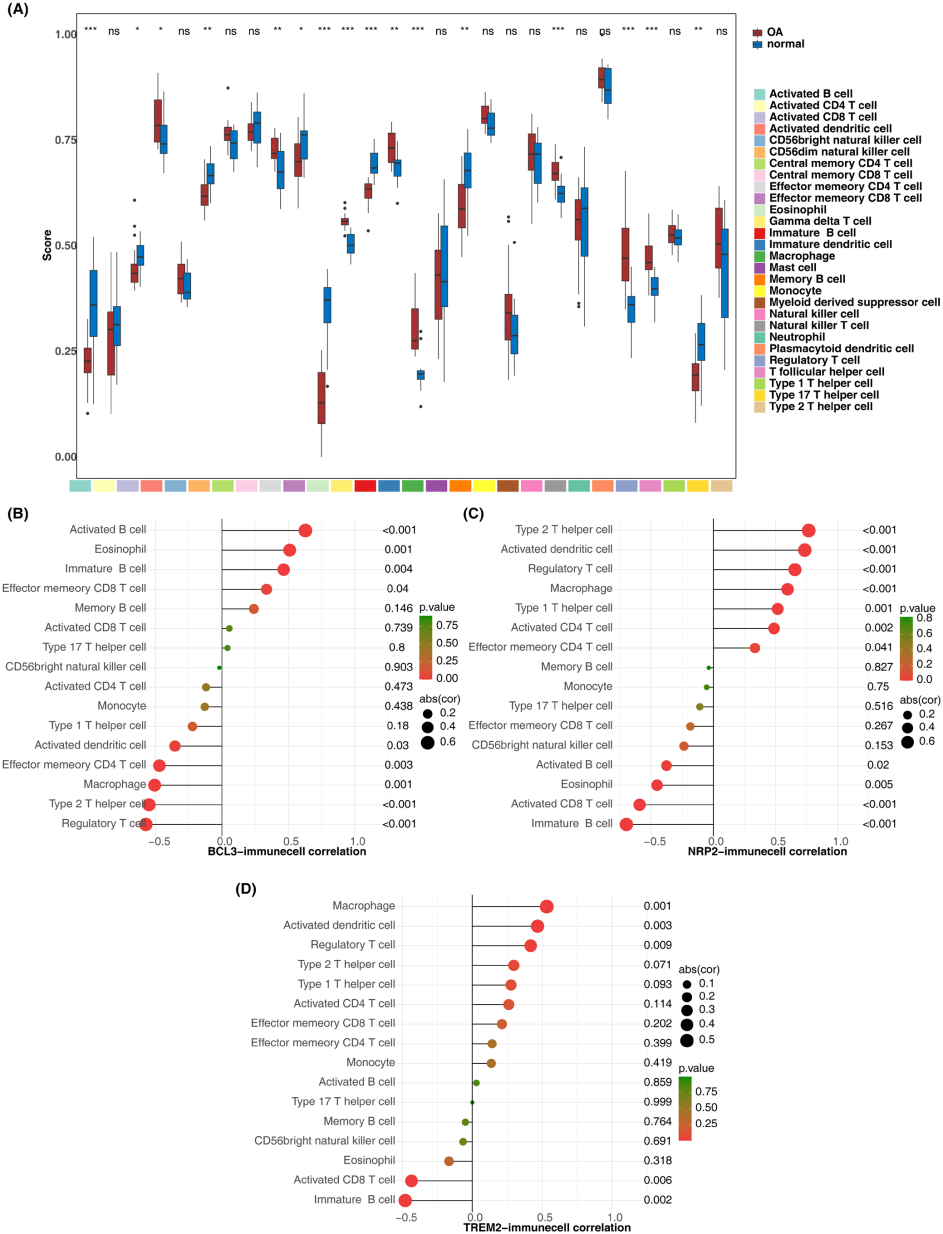
Immune infiltration analysis. (A) Box plots of immune cell infiltration in OA and normal groups. (B–D) Lollipop chart of correlations between biomarkers and differential immune cells.

There were 16 types of immune cells exhibiting obvious differences between the osteoarthritis (OA) and normal groups, including activated B cells, macrophages and regulatory T cells, among others (Figure 4A). Correlation analysis revealed that BCL3 had the strongest positive correlation with activated B cells (*r* = 0.628, *p* < .001) and the strongest negative correlation with regulatory T cells (*r* = −0.572, *p* < .001). NRP2 showed the strongest positive correlation with Type 2 T helper cells (*r* = 0.766, *p* < .001), while TREM2 exhibited the strongest positive correlation with macrophages (*r* = 0.532, *p* < .001). Both NRP2 (*r* = −0.698, *p* < .001) and TREM2 (*r* = −0.481, *p* < .001) displayed the strongest negative associations with immature B cells (Figure 4B–D).

We also predicted the non‐coding RNA regulatory networks for BCL3, TREM2 and NRP2. Six miRNAs (hsa‐miR‐17‐5p, hsa‐miR‐424‐5p, hsa‐miR‐335‐5p, hsa‐miR‐125a‐5p, hsa‐miR‐34a‐5p and hsa‐miR‐196a‐5p) regulating these biomarkers were identified, indicating their potential roles in the regulatory mechanisms underlying OA. Subsequently, upstream lncRNAs were predicted, yielding 261 lncRNAs. Ultimately, a regulatory network was constructed from the three biomarkers (Figure S2A). Moreover, a transcription factor (TF)‐biomarker regulatory network comprising 17 nodes and 16 edges was constructed, in which BCL3 is connected to multiple regulatory relationships, such as with TP53, JUN and SP1 (Figure S2B). Furthermore, a total of 13 candidate drugs with potential therapeutic effects on OA, such as P54, aflibercept and dronedarone, were predicted to target these biomarkers (Figure S2C).

### 3.5 BCL3, NRP2 and TREM2 show differential expression in fibrocartilage chondrocytes (FC) between the OA and normal samples

**FIGURE 5:**
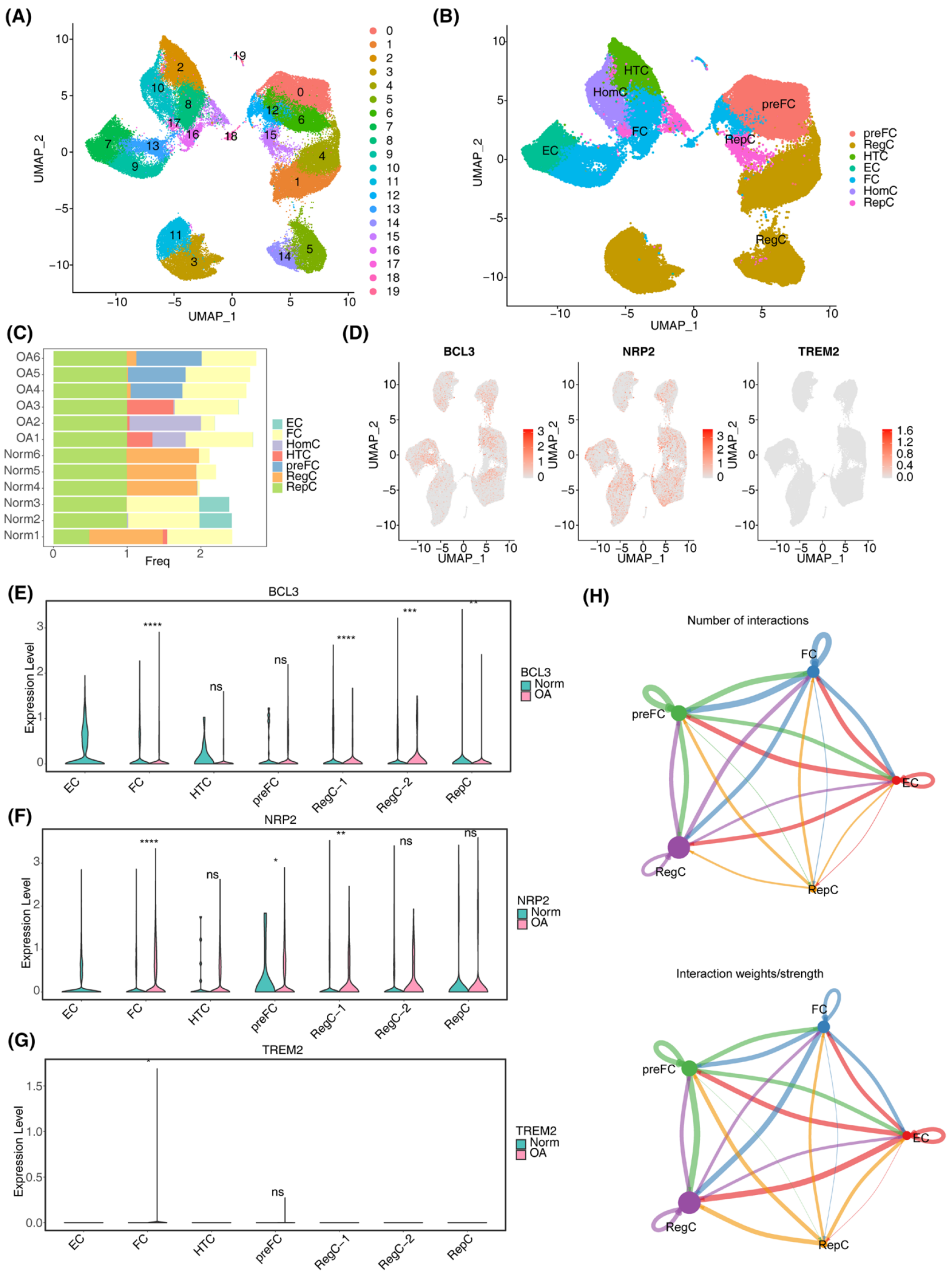
Annotation and analysis of cell types in single cell RNA seq dataset (GSE220243). (A) Visualization of cell clustering. (B) Annotation of cellular subpopulations. (C) Plot of cell proportions for different groups. (D) Expression of biomarkers in different cell populations. (E–G) Expression of biomarkers in different cell populations in different samples. (H) Cellular communication between different cell types.

Single‐cell RNA‐seq data (GSE220243) related to OA was retrieved to analyse the expression of BCL3, NRP2 and TREM2 in different cell populations. After quality control and dimensionality reduction analysis (Figures [Link], [Link], [Link]), the retained cells in GSE220243 were classified into 20 clusters (Figure 5A) and annotated into seven cell types: fibrocartilage chondrocytes (FC), pre‐fibrocartilage chondrocytes (PreFC), regulatory chondrocytes (RegC), hypertrophic chondrocytes (HTC), effector chondrocytes (EC), homeostatic chondrocytes (HomC) and reparative chondrocytes (RepC)^27^ (Figure 5B). The cellular compositions in OA and normal samples are displayed in Figure 5C, with RegC, PreFC and FC accounting for a large proportion in OA samples, and RepC, FC and RegC constituting a significant proportion in normal samples. Figure S6 illustrates the marker genes with the top three significant differences in each cell type. The expression analysis of the biomarkers across different cell types revealed that BCL3 and NRP2 were widely expressed in almost all cell types, while TREM2 was primarily expressed in FC (Figure 5D). Notably, BCL3, NRP2 and TREM2 exhibited significant differences between OA and normal groups in FC population (Figure 5E–G). Regarding cell–cell communication, the number of interactions and interaction weight/strength among PreFC, FC, RegFC and EC were prominent (Figure 5H).

### 3.6 Verification of biomarker expression levels in clinical samples

**FIGURE 6:**
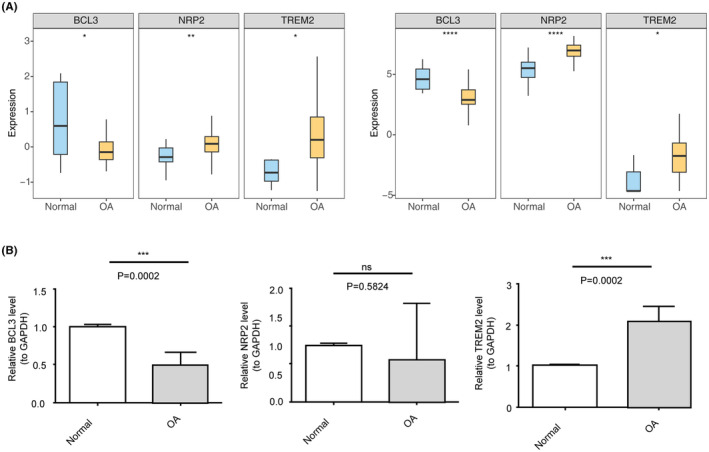
Expression validation of biomarkers. (A) Expression patterns of BCL3, NRP2 and TREM2 in training (left, GSE114007) and validation (right, GSE51588) sets. (B) RT‐qPCR validation of BCL3, NRP2 and TREM2 expression in clinical samples, *n* = 5 samples in each category.

To further validate the expression of biomarkers, the study compared the differences between OA and normal groups in both training and testing datasets. The findings indicated that NRP2 and TREM2 were significantly upregulated in OA samples, whereas BCL3 showed lower expression levels (Figure 6A). Furthermore, to confirm the expression of these key genes, BCL3, NRP2 and TREM2 were selected for RT‐qPCR analysis using clinical samples. The results demonstrated a significant overexpression of TREM2 and low expression of BCL3 in the OA group (Figure 6B). In summary, the RT‐qPCR results aligned closely with those from the public datasets, although we did not find significant difference in NRP2 expression between OA and normal samples.

### 3.7 Downregulation of BCL3 in chondrocytes stimulated by inflammatory cytokines

**FIGURE 7:**
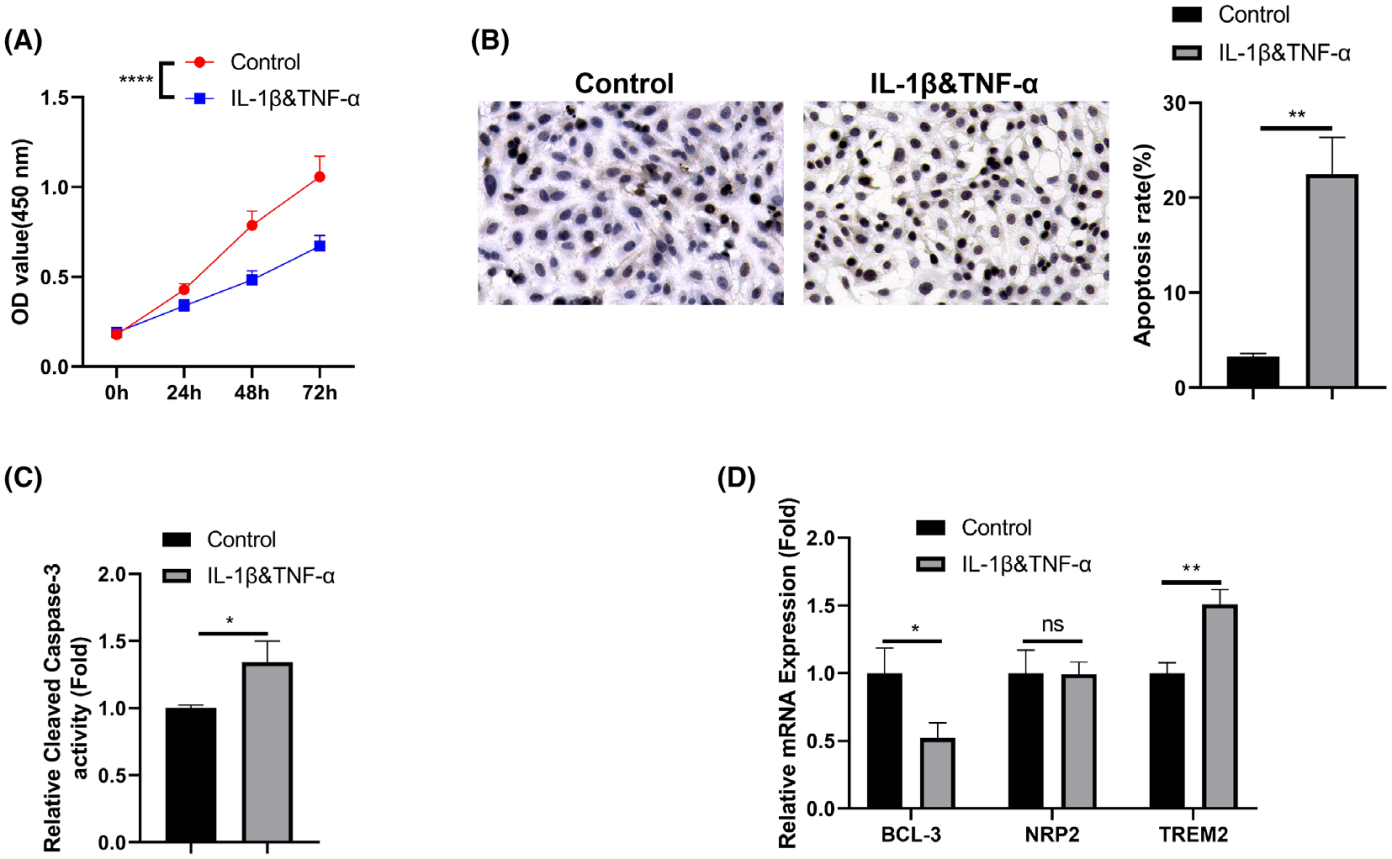
Downregulation of BCL3 in chondrocytes stimulated by inflammatory cytokines. Human chondrocyte cell line (C‐28/I2) was treated with IL‐1β and TNF‐α for 48 h. (A) CCK‐8 cell viability assay; (B) TUNEL staining for assessing apoptotic cell death; (C) Detection of cleaved caspase‐3 activity; (D) RT‐qPCR analysis of BCL3, NRP2 and TREM2 expression. *N* = 3 experiments. *p < .05; **p < .01; ***p < .001; ****p < .0001.

To mimic the inflammatory damages in OA, we treated human chondrocyte cell line (C‐28/I2) with IL‐1β and TNF‐α. Inflammatory stimulation significantly reduced cell viability (Figure 7A). There was also an increase in apoptotic cell death and cleaved caspase‐3 activity after inflammatory stimulation (Figure 7B and C). Furthermore, RT‐qPCR results showed a significant downregulation of BCL3 and an increase of TREM2 in chondrocytes stimulated with IL‐1β and TNF‐α (Figure 7D). These data suggest that BCL3 downregulation may be implicated in the cell death of chondrocytes upon inflammatory stimulation.

### 3.8 BCL3 overexpression rescues chondrocyte cell death induced by inflammatory cytokines

**FIGURE 8:**
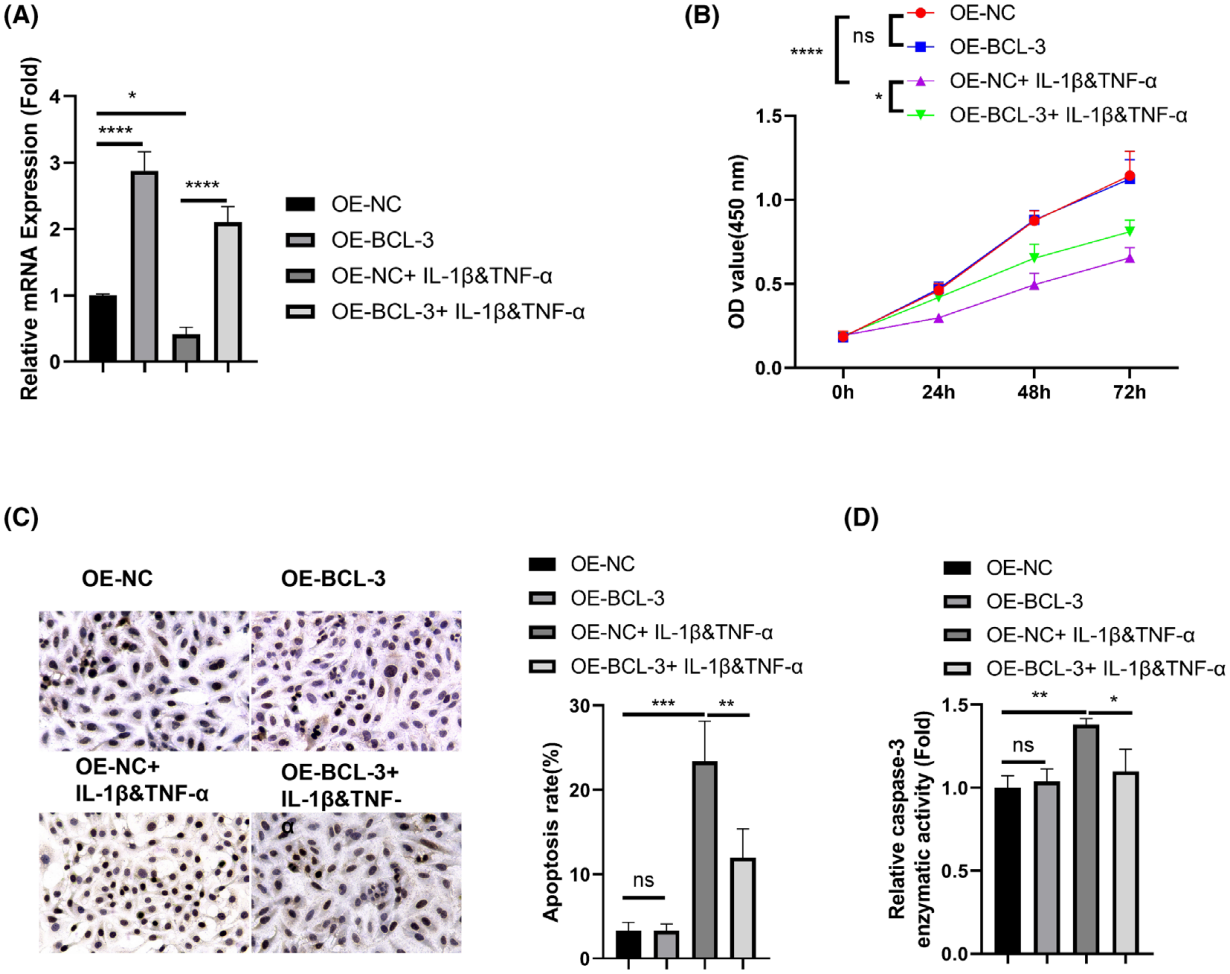
BCL3 overexpression rescues chondrocyte cell death induced by inflammatory cytokines. Human chondrocyte cell line (C‐28/I2) was transfected with pcDNA3.1‐BCL3 to overexpress BCL3 (OE‐BCL3), with the pcDNA3.1 empty vector as the control (OE‐NC). Cells were also treated with IL‐1β and TNF‐α for 48 h. (A) RT‐qPCR analysis of BCL3 in different experimental groups. (B). CCK‐8 cell viability assay; (C) TUNEL staining for assessing apoptotic cell death; (D) Detection of cleaved caspase‐3 activity. *N* = 3 experiments. *p < .05; **p < .01; ***p < .001; ****p < .0001.

To verify the functional engagement of BCL3 in inflammatory cytokine induced cell death, a human chondrocyte cell line C‐28/I2 was transfected with pcDNA3.1‐BCL3 to overexpress BCL3 (OE‐BCL3), with the pcDNA3.1 empty vector as the control (OE‐NC). RT‐qPCR results showed that transfection with pcDNA3.1‐BCL3 successfully increased BCL3 expression in both control and stimulated conditions (Figure 8A). Under inflammatory stimulation, BCL3 overexpression significantly increased cell viability (Figure 8B). The rescue effect was accompanied by reduced cell death as detected by TUNEL staining (Figure 8C), and an decrease in cleaved caspase‐3 activity (Figure 8D). These data further corroborates the suppressive function of BCL3 against inflammation‐induced chondrocyte cell death, indicating a protective role of BCL3 in OA‐related chondrocyte degeneration.

## 4 DISCUSSION

Osteoarthritis is a debilitating condition primarily affecting the knee joint, causing symptoms such as pain, stiffness and limited mobility.^8^ The occurrence and progression of OA are closely linked to the involvement of immune cells and inflammatory factors, particularly in relation to programmed cell death (PCD).^13^ Our research has identified three biomarkers (BCL3, NRP2 and TREM2) exhibiting differential expression in various cell types. To validate these findings, additional tests revealed substantial TREM2 overexpression in OA samples, while BCL3 expression was significantly reduced. These findings indicate that these genes are implicated in OA progression.

BCL‐3 is a member of the IκB family that regulates both apoptosis and immune response.^29^ As a transcriptional coactivator, BCL3 interacts with NF‐κB, particularly p50 and p52 homodimers, modulating NF‐κB‐mediated gene transcription in a context‐dependent manner.^30^, ^31^ In chondrocytes, NF‐κB signalling plays a crucial role in mediating stress responses, including those triggered by pro‐inflammatory cytokines and mechanical stress, which are common in OA conditions.^32^, ^33^ NF‐κB activation in chondrocytes can induce the expression of catabolic enzymes (e.g., MMPs), pro‐inflammatory mediators and pro‐apoptotic factors, all contributing to OA progression.^33^ Our study demonstrates that BCL3 overexpression protects chondrocytes against inflammation‐induced cell death, suggesting that BCL3 may modulate NF‐κB signalling to promote chondrocyte survival under stress. The observed downregulation of BCL3 in OA samples and in chondrocytes upon inflammatory stimulation may indicate a loss of this protective mechanism, potentially exacerbating chondrocyte catabolism and death in OA.^32^ The intricate interplay between BCL3 and NF‐κB signalling in chondrocytes likely represents a delicate balance between pro‐survival and pro‐inflammatory responses, and disruption of this balance could be a key factor in OA pathogenesis. Further research is needed to elucidate the specific mechanisms by which BCL3 interacts with the NF‐κB pathway in chondrocytes under various stress conditions. Furthermore, how BCL3‐NF‐κB interactions affect the expression of genes involved in chondrocyte survival, metabolism and matrix production needs to be clarified.

Neuropilin‐2 (NRP2) is an integral member of the neuropilin protein family, encoded by a gene located at 2q33.3 within the human telomerase‐associated protein family.^34^ It plays a paramount role in critical biological processes, notably cardiovascular development, axon guidance and tumorigenesis, through its interaction with vascular endothelial growth factor (VEGF).^35^ Previous studies have demonstrated increased NRP2 expression in various cancer types, such as lung, colorectal, pancreatic and other tumours.^36^ Research has also revealed that NRP2 expression is elevated in alveolar macrophages (AM) after lung injury, which contributes to pulmonary inflammatory damage.^37^ Since persistent inflammatory damage is a key driving factor in OA pathogenesis,^38^ and it is plausible that the elevation of NRP2 in OA tissue promotes OA development by instigating inflammatory responses. However, it remains to be clarified whether or not NRP2 exacerbates inflammation in OA by targeting macrophages.

TREM2 (Triggering Receptor Expressed on Myeloid Cells 2) is a receptor protein expressed on myeloid cells that regulates immune responses and cell signalling.^39^ TREM2 was reported to regulate the inflammatory activation of microglia (a macrophage in the nervous system) through the phosphatidylinositol‐3‐kinase/protein kinase B/mammalian target of rapamycin (PI3K/Akt/mTOR) signalling pathway,^40^ suggesting the connection between TREM2 and neuroinflammation. Moreover, PI3K/Akt/mTOR signalling pathway is also implicated in cellular apoptosis in OA development.^41^ Therefore, it is plausible that TREM2 could target PI3K/Akt/mTOR signalling in OA and mediate the inflammatory progression, which warrants further exploration.

This study has several limitations that should be considered. First, while we validated some findings using RT‐qPCR on clinical samples, the sample size was relatively small. Larger cohorts are needed to further confirm the clinical relevance of the identified biomarkers. Further, our functional studies focused on BCL3 in an in vitro chondrocyte model, which may not fully recapitulate the complex in vivo environment of OA joints. Future studies using animal models and human tissue samples are warranted to further elucidate the roles of the identified biomarkers in OA pathogenesis. Additionally, non‐coding RNAs, such as miRNAs, are crucial gene regulators in cartilage homeostasis and OA progression.^41^, ^42^ Our study also revealed a regulatory network of non‐coding RNAs related to BCL3, NRP2 and TREM2. Understanding the regulation patterns of miRNAs and lncRNAs may offer novel perspectives on the manipulation of BCL3, NRP2 and TREM2 for delaying the progression of OA.

## 5 CONCLUSION

To sum up, we identified BCL3, TREM2 and NRP2 as potential biomarkers for programmed cell death mechanisms in OA progression through integrated bioinformatics analyses of multiple datasets. BCL3 exhibited anti‐apoptotic effects in an inflammatory chondrocyte model. The biomarkers were associated with ribosome function, immune infiltration and regulatory networks involving non‐coding RNAs and transcription factors. The findings provide insights into OA biology and support further investigation of these genes as predictive biomarkers and therapeutic targets regulating programmed cell death pathways in OA. Future research should validate the biomarkers clinically and investigate the functional mechanisms through in vivo models.

## AUTHOR CONTRIBUTIONS

All authors were involved in the conceptualization and design of the study. Yongqing Xu provided experimental ideas. Junxiao Ren and Rui LI completed the first draft of the manuscript. Chen Meng performed the material preparation, data collection and analysis. Chuan Li was mainly responsible for reviewing the final manuscript. All authors participated in the editorial revision of the manuscript. All authors read and approved the final manuscript.

## FUNDING INFORMATION

This work was supported by Yunnan Orthopedics and Sports Rehabilitation Clinical Medical Research Center (202102AA310068). General Project of Yunnan Province Kunming Medical Joint Special Basic Research Program (202001AC070337). Yunnan Province technology innovation talent training object project (202005AD160146).

## CONFLICT OF INTEREST STATEMENT

The authors declare that they have no competing interests.

## ETHICS STATEMENT

The use of human samples gained the approval of the 920th Hospital Ethics Committee of the Joint Logistic Support Force (No.2023–124‐01).

## CONSENT FOR PUBLICATION

All authors and subjects were informed and consented to publication.

## Supporting information


**Figure S1.** Identification and analysis of DE‐PMRGs. **(a)** Venn plot of DE‐PMRGs. **(b)** Tree diagram of GO, KEGG enrichment analysis of DE‐PMRGs (top 10).

**Figure S2.** Regulatory mechanisms of biomarkers. (a) The lncRNA‐miRNA‐mRNA regulatory network. (b) TF‐biomaker regulatory network. (c) Biomarker‐drug prediction network diagram.

**Figure S3.** Single‐cell data quality control chart.

**Figure S4.** Screening for highly variable genes.

**Figure S5.** Principal component fragmentation chart.

**Figure S6.** Marker genes spot map in each cell types.

## Data Availability

The datasets generated during and/or analysed during the current study are available from the corresponding author upon reasonable request.
