# Supplementary figures and images for: Identification of BCL3 as a biomarker for chondrocyte programmed cell death in osteoarthritis
Source: Int J Exp Pathol. 2024 Dec 16;106(1):e12522. doi: 10.1111/iep.12522 (PMC11731105; doi:10.1111/iep.12522)

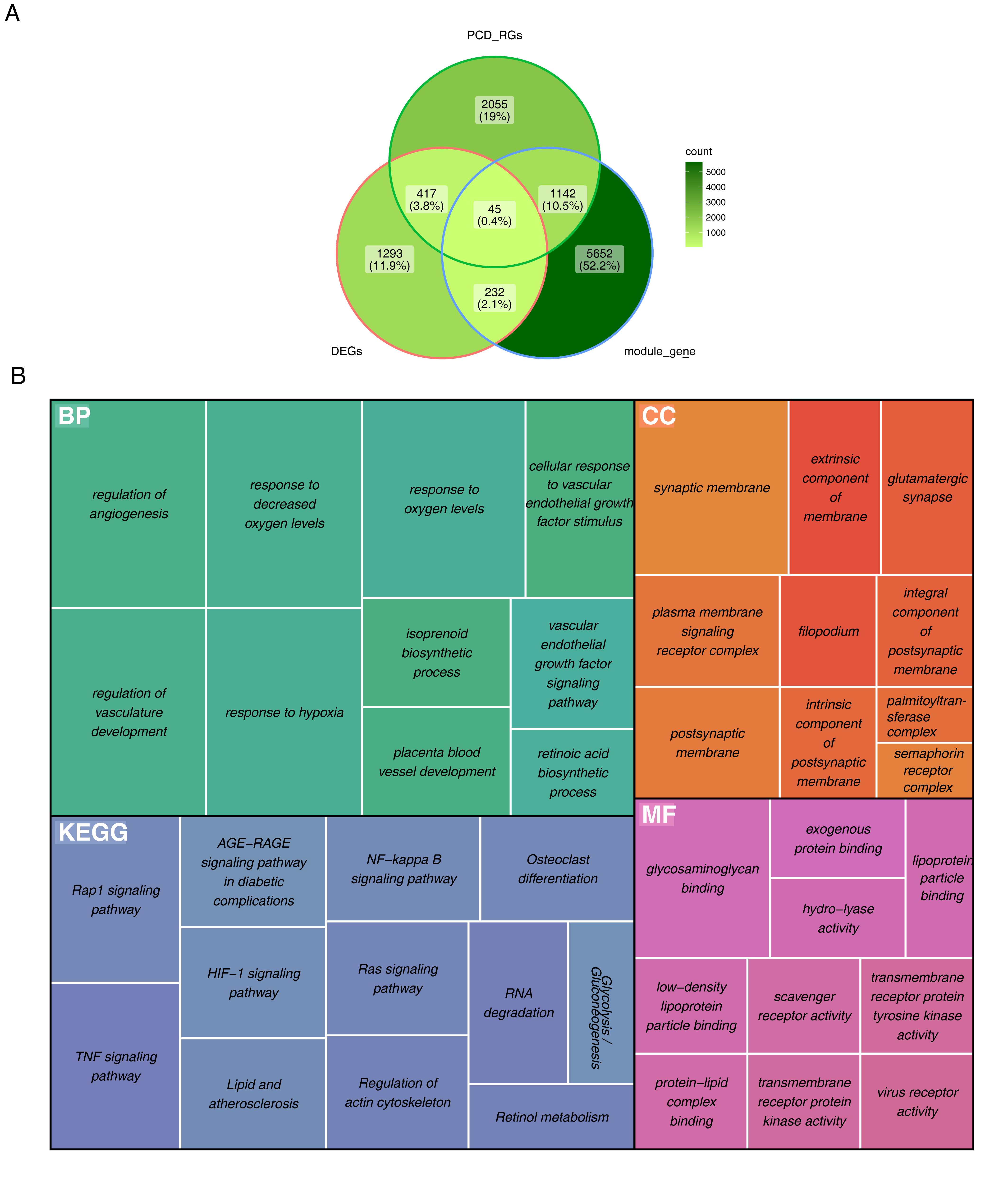

Supplement: Supplementary file 1 — Figure S1. Identification and analysis of DE‐PMRGs. (a) Venn plot of DE‐PMRGs. (b) Tree diagram of GO, KEGG enrichment analysis of DE‐PMRGs (top 10). [file IEP-106-e12522-s002.tif]

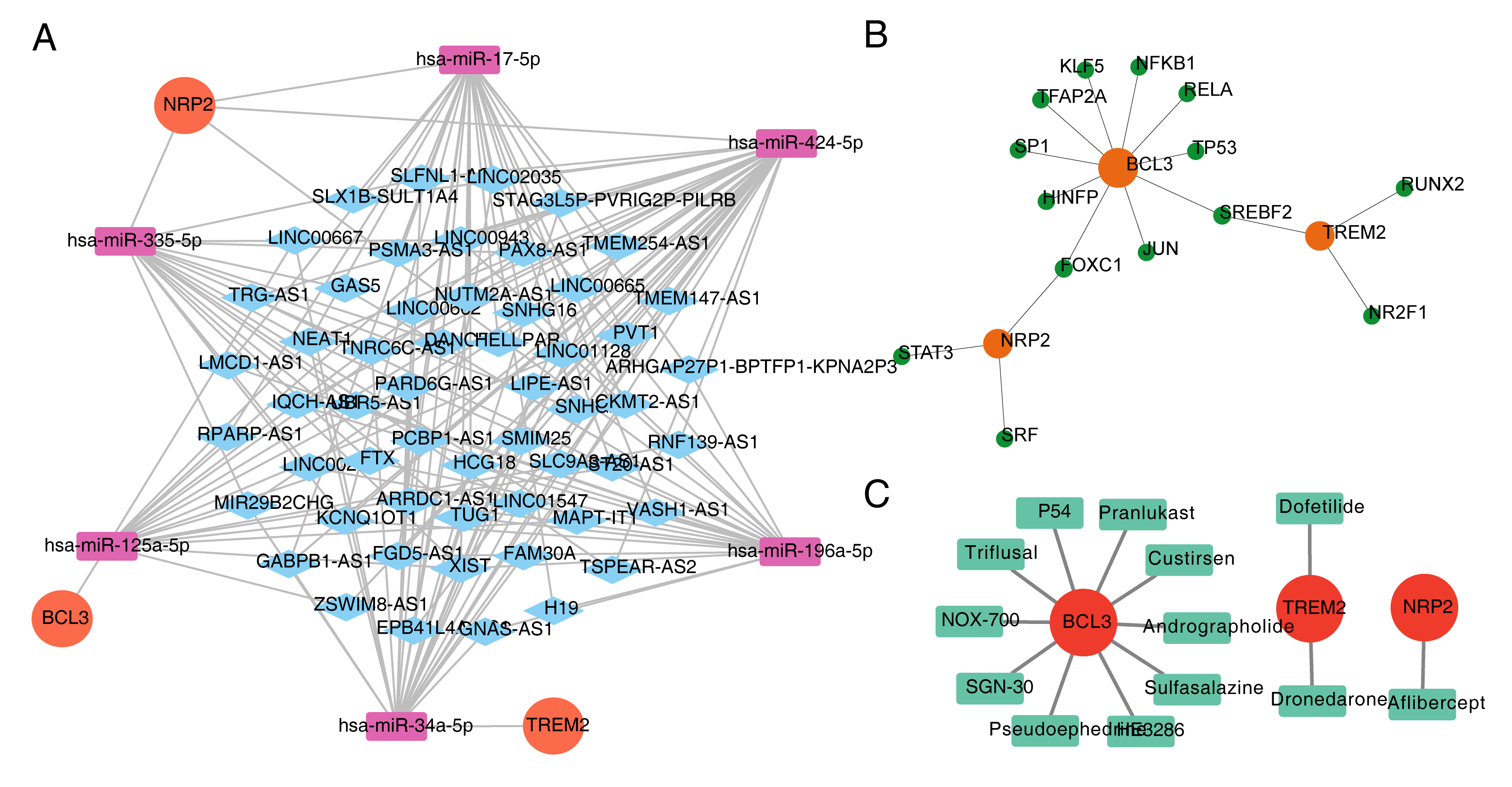

Supplement: Supplementary file 2 — Figure S2. Regulatory mechanisms of biomarkers. (a) The lncRNA‐miRNA‐mRNA regulatory network. (b) TF‐biomaker regulatory network. (c) Biomarker‐drug prediction network diagram. [file IEP-106-e12522-s003.tif]

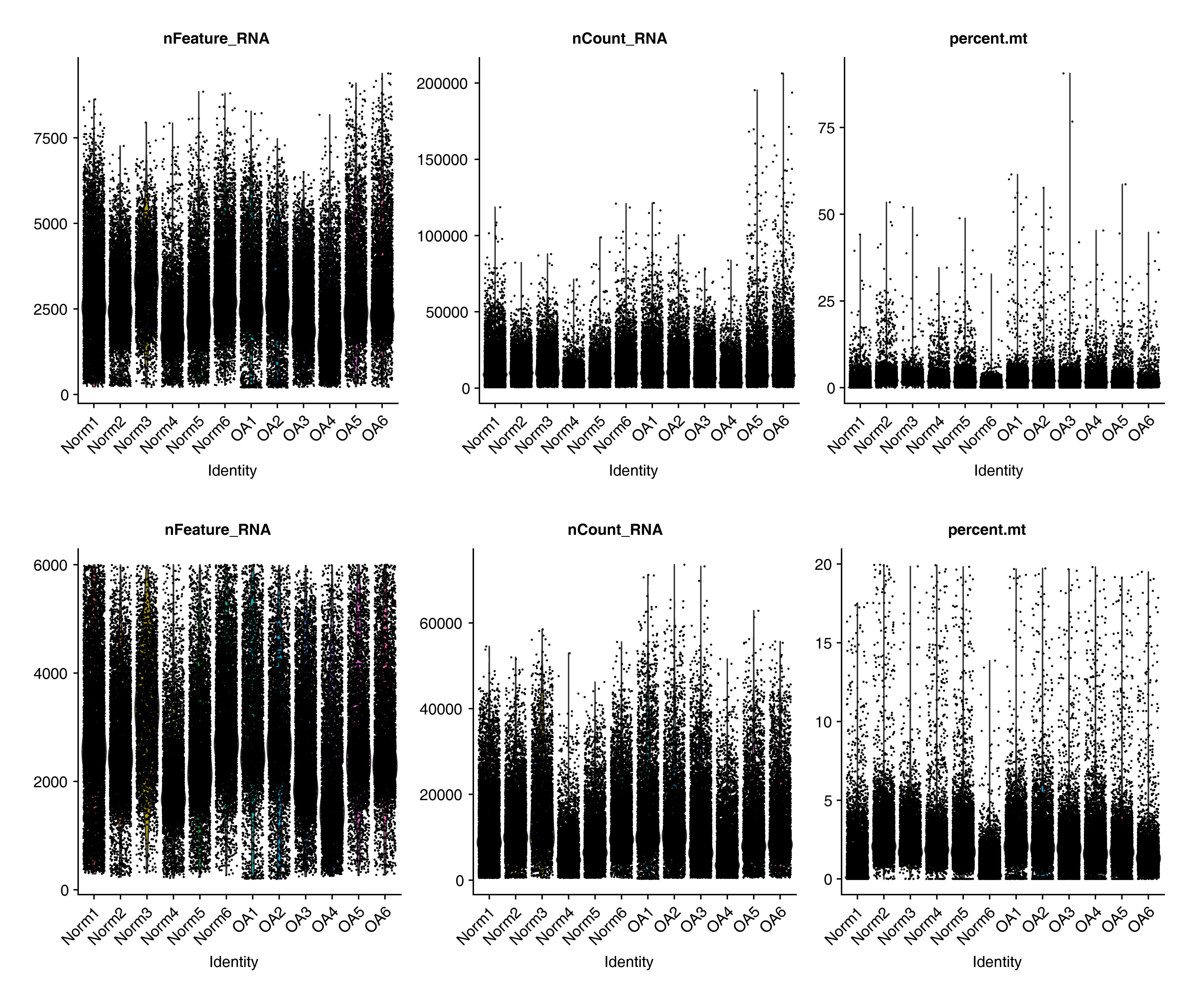

Supplement: Supplementary file 3 — Figure S3. Single‐cell data quality control chart. [file IEP-106-e12522-s005.tif]

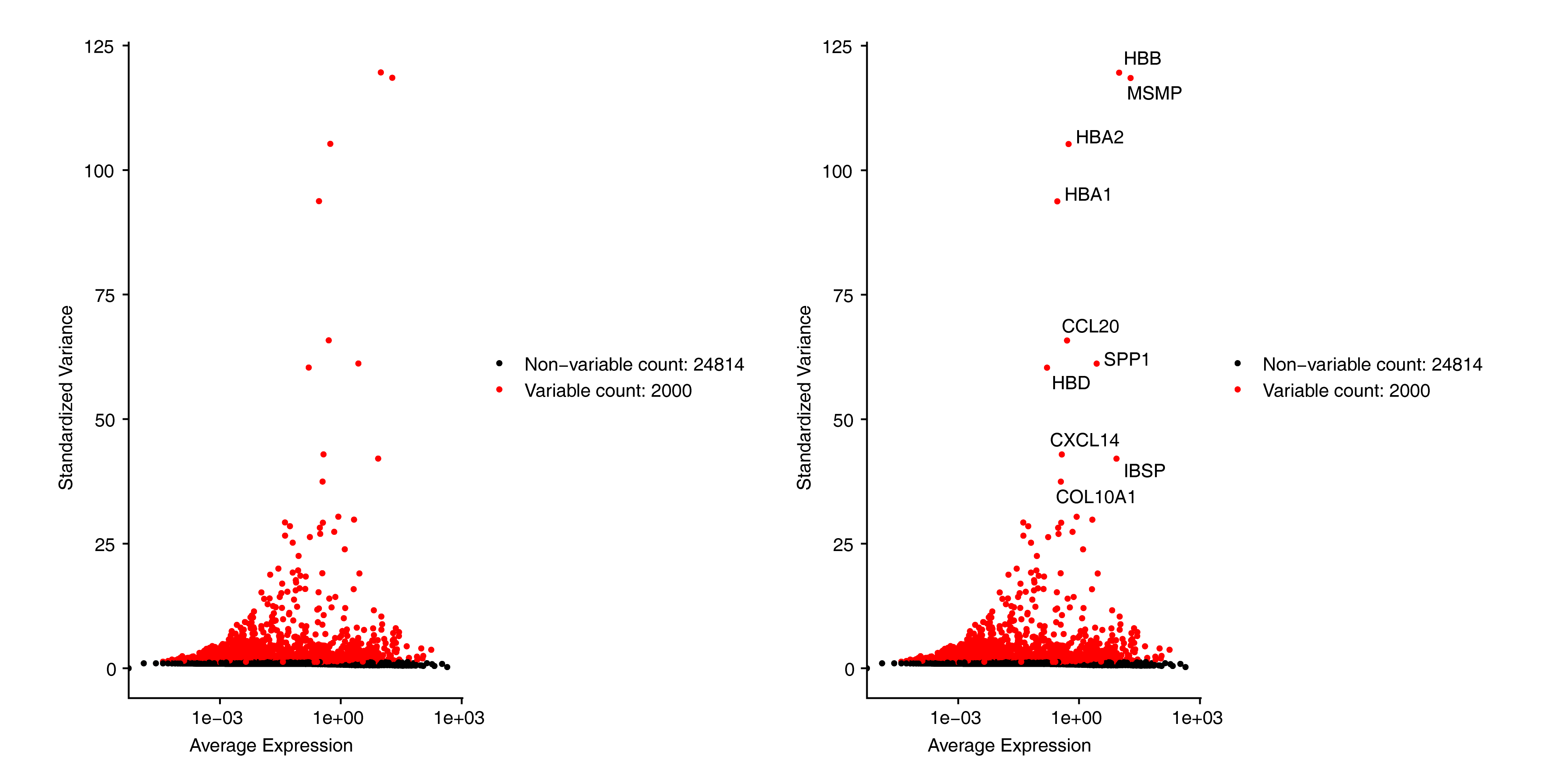

Supplement: Supplementary file 4 — Figure S4. Screening for highly variable genes. [file IEP-106-e12522-s006.tif]

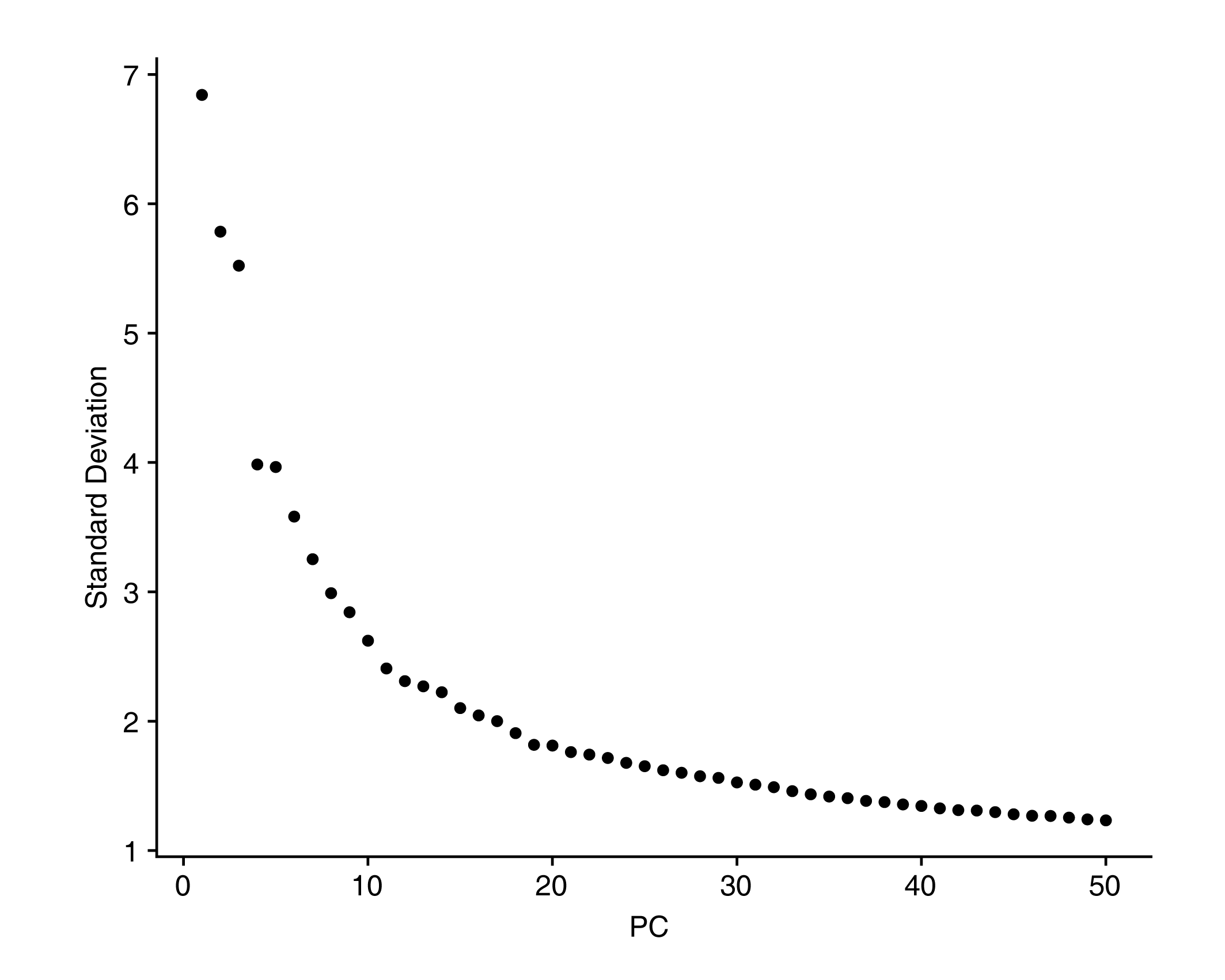

Supplement: Supplementary file 5 — Figure S5. Principal component fragmentation chart. [file IEP-106-e12522-s001.tif]

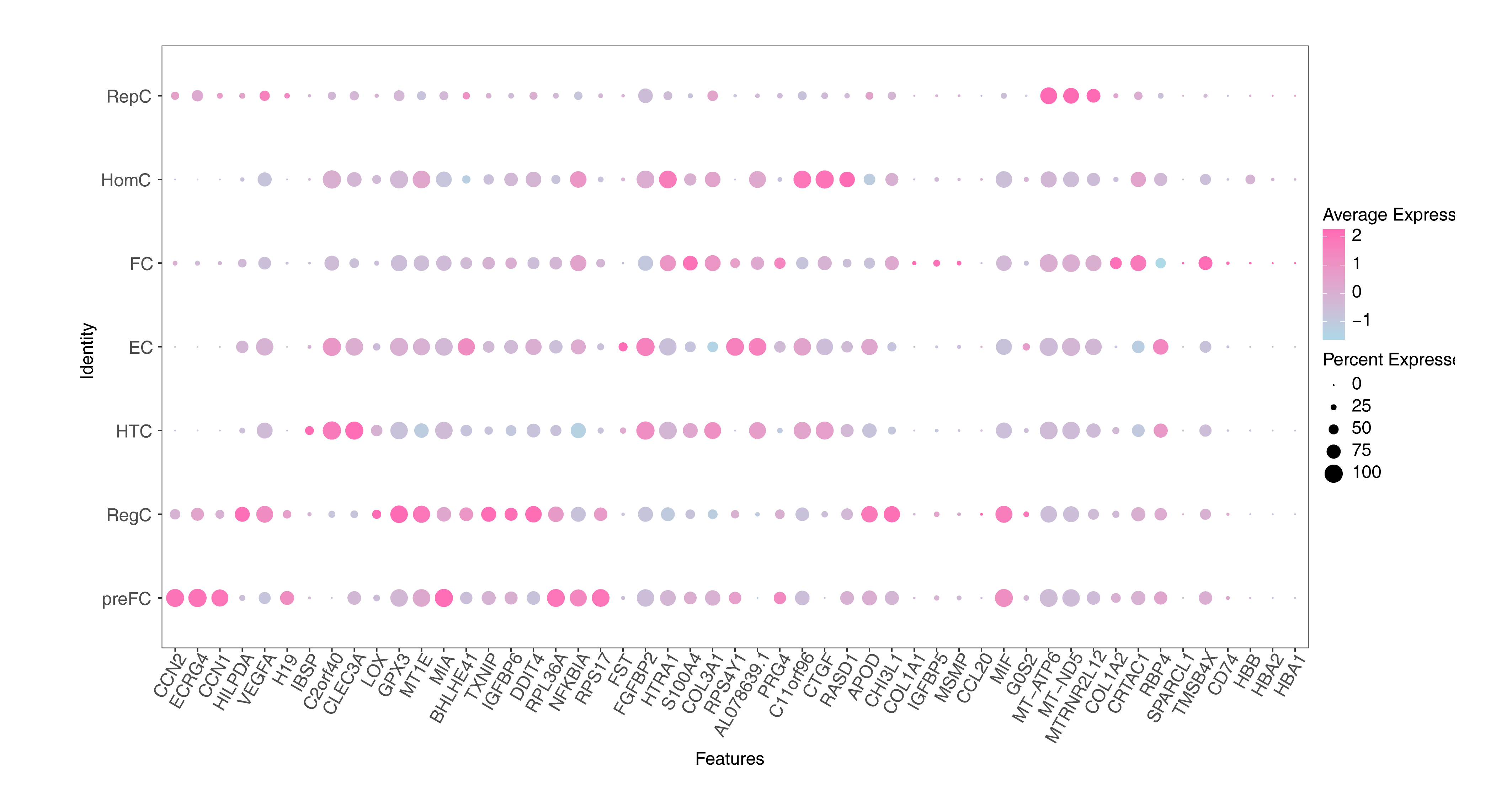

Supplement: Supplementary file 6 — Figure S6. Marker genes spot map in each cell types. [file IEP-106-e12522-s004.tif]
